# Prodrugs for Skin Delivery of Menahydroquinone-4, an Active Form of Vitamin K_2(20)_, Could Overcome the Photoinstability and Phototoxicity of Vitamin K_2(20)_

**DOI:** 10.3390/ijms20102548

**Published:** 2019-05-24

**Authors:** Shotaro Goto, Shuichi Setoguchi, Hirofumi Yamakawa, Daisuke Watase, Kazuki Terada, Kazuhisa Matsunaga, Yoshiharu Karube, Jiro Takata

**Affiliations:** Faculty of Pharmaceutical Sciences, Fukuoka University, Nanakuma, Jonan-ku, Fukuoka 814-0180, Japan; sgoto@fukuoka-u.ac.jp (S.G.); ssetoguchi@fukuoka-u.ac.jp (S.S.); hyamakawa@adm.fukuoka-u.ac.jp (H.Y.); watase@fukuoka-u.ac.jp (D.W.); kterada@fukuoka-u.ac.jp (K.T.); karube@fukuoka-u.ac.jp (Y.K.)

**Keywords:** menaquinone-4, menahydroquinone-4, prodrug, drug delivery system, phototoxicity, photostability, photodegradation, skin application, vitamin K

## Abstract

The effective delivery of menahydroquinone-4 (MKH), an active form of menaquinone-4 (MK-4, vitamin K_2(20)_), to the skin is beneficial in the treatment of various skin pathologies. However, its delivery through the application of MK-4 to the skin is hampered due to the photoinstability and phototoxicity of MK-4. This study aimed to evaluate the potential of ester prodrugs of MKH for its delivery into the skin to avoid the abovementioned issues. The ester prodrugs, MKH 1,4-bis-*N*,*N*-dimethylglycinate hydrochloride (MKH-DMG) and MKH 1,4-bis-hemisuccinate (MKH-SUC), were prepared using our previously reported methods. Photostability was determined under artificial sunlight and multi-wavelength light irradiation, phototoxicity was determined by intracellular ROS formation and cell viability of UVA-irradiated human epidermal keratinocyte cells (HaCaT), and delivery of MKH into HaCaT cells was assessed by measuring menaquinone-4 epoxide (MKO) levels. MKH prodrugs showed higher photostability than MK-4. Although MK-4 induced cellular ROS and reduced cell viability after UVA irradiation, MKH prodrugs did not affect either ROS generation or cell viability. MKH prodrugs enhanced intracellular MKO, indicating effective delivery of MKH and subsequent carboxylation activity. In conclusion, these MKH prodrugs show potential for the delivery of MKH into the skin without photoinstability and phototoxicity.

## 1. Introduction

Menaquinone-4 (MK-4) or vitamin K_2(20)_ is an important vitamin K compound used clinically. Menahydroquinone-4 (MKH), the fully reduced and active form of MK-4, is a cofactor for γ-glutamyl carboxylase (GGCX), which catalyzes the post-transcriptional carboxylation of vitamin K-dependent proteins. MKH is produced by the enzymatic conversion of vitamin K_1_ and K_3_ in the body [1]. Since MKH is readily oxidized to MK-4, MK-4 is available for clinical use.

Skin application of vitamin K exhibits several beneficial effects such as suppressing pigmentation and resolving bruising [2,3,4], prophylactically limiting the occurrence of acneiform side effects in patients receiving the monoclonal antibody cetuximab [5,6,7,8], and promoting wound healing [9].

Because vitamin K is highly unstable in light, it should be strictly protected from light exposure during manufacturing, preparation, storage, and treatment [10,11,12]. Furthermore, vitamin K is phototoxic against cultured epidermis upon ultraviolet A (UVA) irradiation (dose 6 J/cm^2^) [13]. Given that when vitamin K is applied to exposed skin, it is difficult to protect it from light exposure, and these physicochemical properties of MK-4 limit its use as a skin application and hamper its beneficial effects.

**Figure 1:**
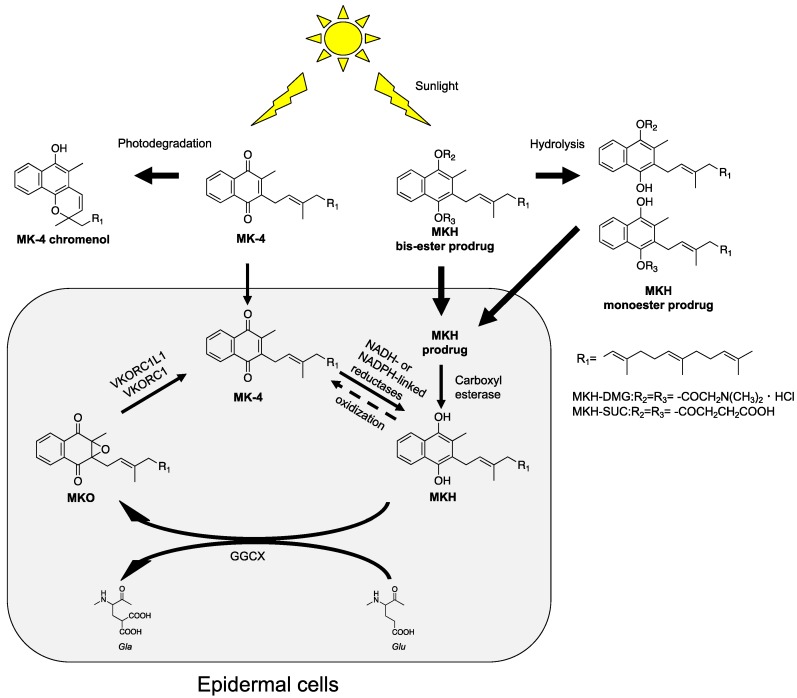
Concept of dermal delivery of MKH that avoids photoinstability and phototoxicity using a prodrug approach. Solid line: the advocated or expected processes based on the literatures and obtained results in this study. Dashed line: the possible process during the drug measurement.

It has been shown that the main photodegradation product of MK-4 is MK-4 chromenol (MKC), which is produced from the naphthoquinone structure and isoprenyl side chain of MK-4. We have previously reported that ester-type prodrugs of MKH, such as the 1,4-bis-*N*,*N*-dimethylglycinate (MKH-DMG) and hemi-succinate (MKH-SUC) derivatives, can act as delivery systems for MKH without a reductive activation step [14,15,16,17]. Since these compounds do not contain the naphthoquinone structure, these prodrugs may overcome the photoinstability and phototoxicity problems. It has been reported that the partition coefficient (log P) of MKH-DMG at physiological pH 7.4 is 3.66 [14]; this value indicates that MKH-DMG has good lipophilicity for membrane permeation. In addition, MKH-DMG and MKH-SUC could deliver MKH into hepatocellular carcinoma (HCC) cells more effectively than MK-4 [17]. Thus, MKH-DMG and MKH-SUC were considered to be good candidate compounds for skin application. In the current study, we examined their effectiveness as photostable and low-phototoxicity prodrugs of MKH in order to further development of a vitamin K skin application. The concept underlying the dermal delivery system of MKH is shown in Figure 1.

## 2. Results

### 2.1. Photostability of MKH-Ester Derivatives in Artificial Sunlight

**Figure 2:**
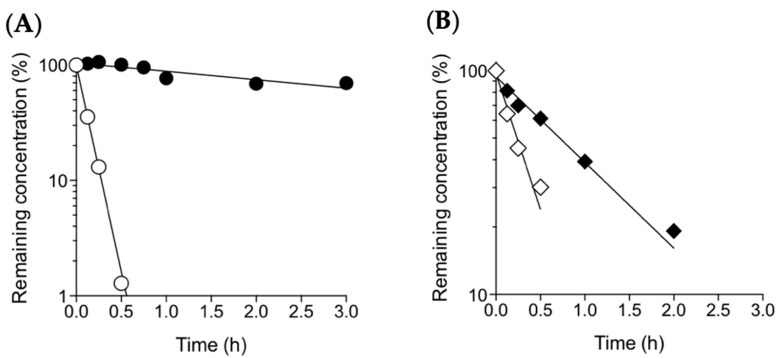
Photostability of MK-4 and MKH derivatives (1 µM in ethanol) in artificial sunlight (12,000 lx) at 25 °C. (**A**) ○: MK-4 and ●: MKH-DMG; (**B**) MKH-SUC with (◆) and without (◇) shading. Remaining concentration was calculated as a percentage of the initial concentration of each respective compound.

To evaluate the photostability of the MK-4 and MKH derivatives in skin application, 1 μM solutions of MK-4, MKH-DMG, or MKH-SUC in ethanol were irradiated with artificial sunlight (12,000 lx) at 25 °C with and without shading, and the final amounts of MKH derivatives and their monoesters, MK-4 and MK-4 chromenol, were determined by LC-MS/MS. The relative irradiance of artificial sunlight at wavelengths of 300 to 800 nm is almost equal to that of natural sunlight, according to the manufacturer. MK-4 and MKH-DMG concentrations decreased in sunlight irradiation according to an apparent first order rate (Figure 2A), but were unchanged under shading. MKH-SUC concentration decreased according to an apparent first order rate both with and without shading, although the rate of decrease was accelerated without shading. Apparent first order constants and half-lives are listed in Table 1. MKH-DMG and MKH-SUC were more photostable than MK-4 by ca. 50- and 3-fold, respectively. These results indicate that the use of MKH ester derivatives is a valid method to overcome the photolability of MK-4. In particular, MKH-DMG exhibits excellent photostability in potential skin application.

Following sunlight irradiation, MK-4 in ethanol was mainly converted to MK-4 chromenol. This photochemical result is consistent with previously reported results in polar solvents [11,12]. In contrast, MKH-DMG was mainly hydrolyzed to the corresponding monoester (MKH-mono-DMG). MKH-SUC was hydrolyzed to MKH-mono-succinate (MKH-mono-SUC) under shading, with this hydrolytic conversion being accelerated by unshaded sunlight irradiation (Figure 2B) (Chromatograms of MK-4 chromenol, MKH-mono-DMG, and MKH-mono-SUC are shown in Supplemental Figure S1). These results suggest that the difference in the degree of hydrolysis of MKH-DMG and MKH-SUC at shading is probably due to the instability of the ester bonds.

**Figure 3:**
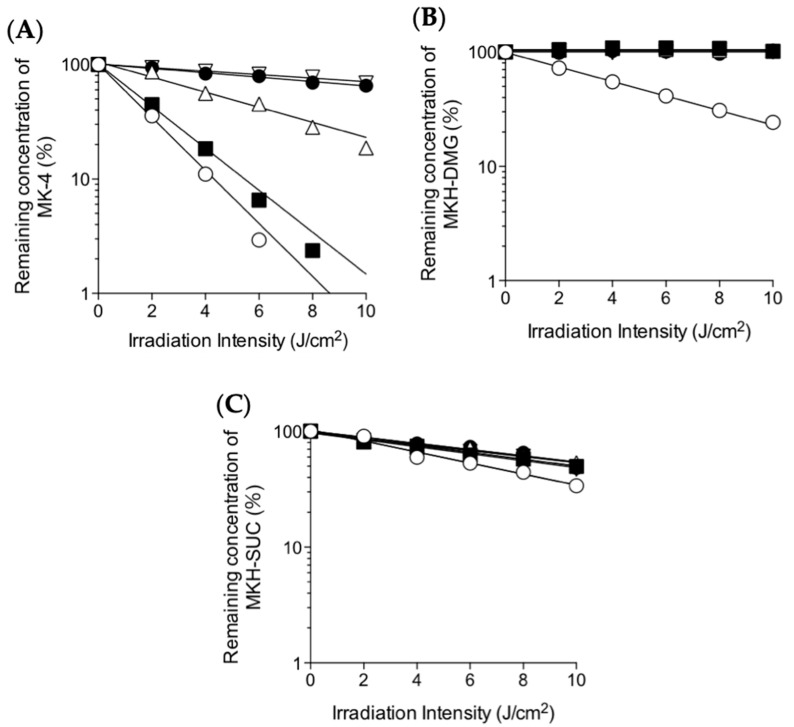
Effects of irradiation wavelength on the photodegradation of MK-4 and MKH derivatives at 25 °C. (**A**) MK-4; (**B**) MKH-DMG; and (**C**) MKH-SUC were irradiated with monochromatic light of wavelengths ○: 279, ■: 341, △: 373, ●: 404, and ▽: 435 nm. Remaining concentration was calculated as a percentage of the initial concentration of each respective compound.

To evaluate the effect of irradiation wavelength on photostability of MK-4 and MKH-ester derivatives, 1 μM solutions of MK-4, MKH-DMG, and MKH-SUC in ethanol were irradiated with a xenon light source monochromatic light at 279, 341, 373, 404, or 435 nm, and the concentration of each compound was measured by LC-MS/MS. The log of concentration of each compound was plotted against irradiation energy (Figure 3), and the rates and half-life energies of degradation were obtained from slopes (Table 2). MK-4 decomposition, primarily due to MK-4 chromenol, was accelerated by a wide range of wavelengths (279−435 nm, Figure 3A). Hydrolytic degradations of MKH-DMG and MKH-SUC to their corresponding monoesters were accelerated only at the short wavelength of 279 nm (Figure 3B,C).

### 2.2. Phototoxicity of MK-4 and MKH-Ester Derivatives

Some pharmaceutical substances are photosensitizers that can lead to the generation of reactive oxygen species (ROS) that cause oxidative damage to skin cells. It has been also shown that vitamin K_1_ exhibits phototoxicity against cultured epidermis upon UVA irradiation [13]. Therefore, we evaluated the generation of ROS from MK-4 and MKH derivatives by UVA (320–400 nm) irradiation, which is strongly related to phototoxicity and accounts for approximately 95% of total ultraviolet radiation in sunlight that reaches the surface of the earth.

**Figure 4:**
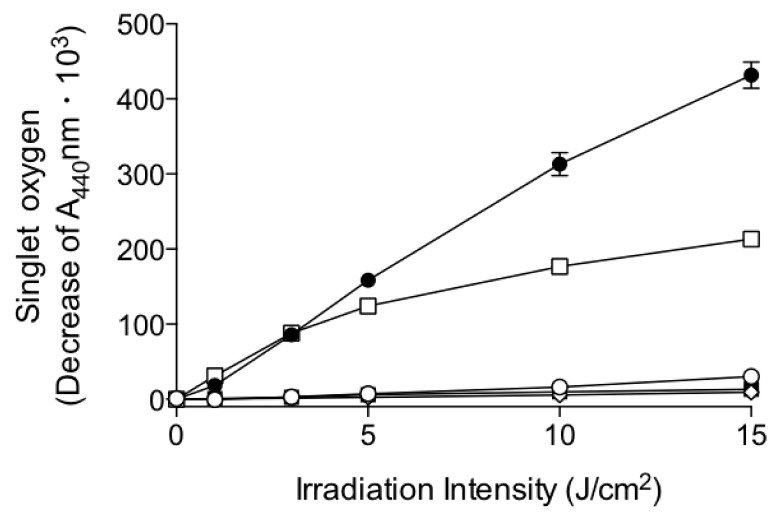
Effect of UVA irradiation intensity on singlet oxygen generation in aqueous solutions of MK-4 and MKH derivatives. □: MK-4, ■: MKH-DMG, ○: MKH-SUC, ●: ketoprofen (positive control), and ◇: sulisobenzone (negative control). Data represent mean ± SD (*n* = 3).

It has been reported that singlet oxygen promotes the peroxidation of skin surface lipids, resulting in the induction of skin inflammation [18]. Thus, we measured the irradiation intensity-dependent generation of singlet oxygen by MK-4, MKH-DMG, and MKH-SUC in phosphate-buffered aqueous solution after UVA irradiation using a *p*-nitrosodimethylaniline-based ROS assay (Figure 4). Following UVA irradiation (0–15 J/cm^2^), MK-4 exhibited increased formation of singlet oxygen in an irradiation energy-dependent manner similar to that of ketoprofen, the positive control, whereas MKH-DMG and MKH-SUC showed little generation of singlet oxygen, comparable to that of sulisobenzone, the negative control (Figure 4). The results indicate that the quinone structure of MK-4 accelerates singlet oxygen generation by UVA irradiation and that the singlet oxygen formation of MKH ester derivatives is unaffected.

**Figure 5:**
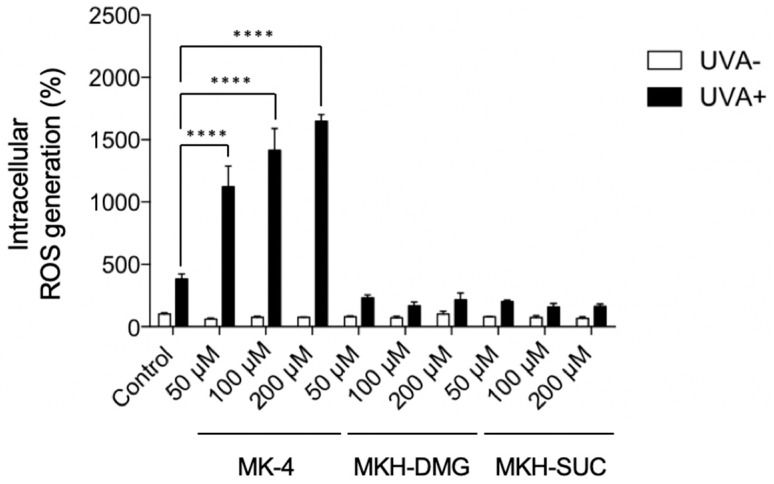
Intracellular ROS generation in HaCaT cells treated with MK-4 and MKH-ester derivatives and irradiated with UVA. HaCaT cells were treated with MK-4, MKH-DMG, or MKH-SUC in the presence or absence of UVA irradiation (15 J/cm^2^). Intracellular ROS generation was measured using a DCFH-DA assay. Data represent mean ± SD (*n* = 3). **** *p* < 0.001 by Dunnett’s test.

**Figure 6:**
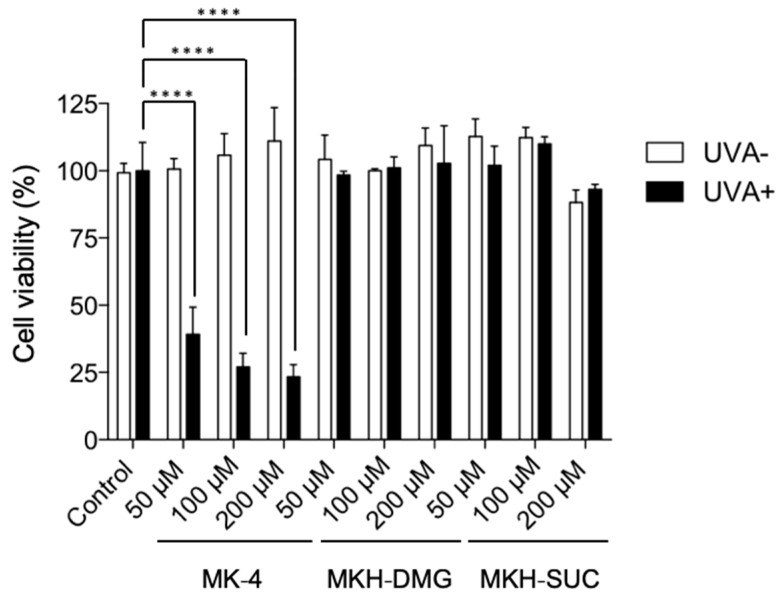
Viability of HaCaT cells treated with MK-4 and MKH-ester derivatives and irradiated with UVA. Cell viability was assessed at 24 h after UVA irradiation (15 J/cm^2^). Data represent mean ± SD (*n* = 3). **** *p* < 0.001 by Dunnett’s test.

The in vitro phototoxicity of MK-4 and MKH derivatives were assessed by measuring the intracellular ROS generation and the viability of human epidermal keratinocyte cells (HaCaT) after UVA irradiation in the presence or absence of MK-4 and MKH derivatives. Intracellular ROS generation was determined using the cell permeable probe 2′,7′-dichlorofluorescein diacetate (DCFH-DA), which rapidly reacts with hydroxyl radicals. Cells without drug treatment or UVA irradiation were used as the control. ROS generation and cell viability are shown in Figure 5 and Figure 6, respectively.

None of the compounds increased intracellular ROS levels in the absence of UVA (Figure 5). Following UVA irradiation, the intracellular ROS level in the MK-4 treated group was elevated in a dose-dependent manner, whereas no significant changes in intracellular ROS levels were observed in the MKH-DMG and MKH-SUC treated groups (Figure 5).

None of the compounds affected cell viability in the absence of UVA irradiation (Figure 6). After UVA irradiation, cells were re-incubated for 24 h and analyzed for cell viability. The viability of MK-4-treated cells was decreased dose-dependently (Figure 6), while the viability of cells treated with MKH-DMG or MKH-SUC was unaffected (Figure 6). Increased intracellular ROS and decreased cell viability were only observed in cells treated with MK-4 and irradiated with UVA, suggesting that the phototoxicity of MK-4 was a result of ROS formation. Because DCFH-DA mainly reacts with highly reactive ROS such as hydroxyl radicals and peroxynitrate, cell toxicity may be caused by ROS and/or induced H_2_O_2_.

Based on these results, it appears that MKH derivatives would be useful for skin application without producing singlet oxygen related skin surface toxicity and ROS related cytotoxicity induced by photo irradiation.

### 2.3. MKH Delivery into HaCaT Cells Using MK-4 and MKH-Ester Derivatives

The potential of MKH derivatives and MK-4 as dermal delivery systems of MKH were assessed by measuring intracellular MKH levels. However, accurate measurement of MKH is difficult because MKH is readily oxidized to MK-4. In γ-glutamyl carboxylation of vitamin K dependent protein (VKDP), MKH acts as a cofactor of γ-glutamyl carboxylase (GGCX) and is stoichiometrically converted to menaquinone-4 epoxide (MKO). Thus, MKO levels in HaCaT cells can reflect the amount of MKH delivered into the cell and subsequently converted in the carboxylation of VKDP. In addition, MK-4 level in cells after MKH prodrugs treatment indicates the oxidative product of MKH.

**Figure 7:**
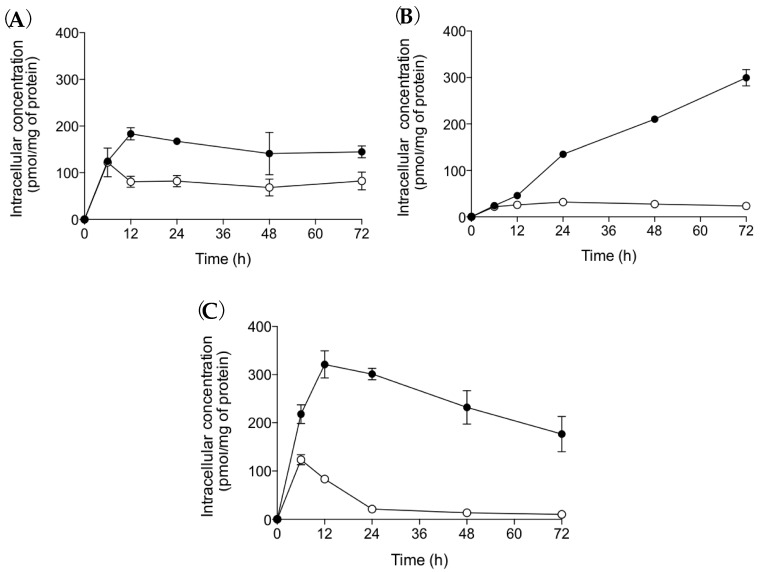
MKO and MK-4 levels in HaCaT cells after treatment with MK-4 or MKH derivatives. HaCaT cells were treated with (**A**) MK-4, (**B**) MKH-DMG, or (**C**) MKH-SUC (5 µM). The intracellular concentrations of ●: MKO, and ○: MK-4 were determined by LC-MS/MS. Data represent mean ± SD (*n* = 3).

In this study, the intracellular concentration of MKO and MK-4 in HaCaT cells over time after treatment with MK-4, MKH-DMG, or MKH-SUC (5 μM) was determined by LC-MS/MS (Figure 7).

After MK-4 treatment, MKO and MK-4 levels immediately increased and reached a plateau after 12 to 24 h. Following MKH-DMG and MKH-SUC treatment, MKO levels were significantly increased, but the increase in MKO levels with MKH-SUC was faster than that with MKH-DMG. The areas under the concentration vs time curves over 72 h for MKO (AUC_MKO (0–72 h)_) and MK-4 (AUC_MK-4 (0–72 h)_) were calculated using the trapezoidal method and are shown in Table 3. The AUC_MKO (0–72 h)_ for MKH-DMG and MKH-SUC was 1.023- and 1.641-fold that of MK-4, respectively. These results indicate that MKH-DMG and MKH-SUC can act as MKH prodrugs and effectively deliver MKH to keratinocytes.

The time course of intracellular MKO depends on the cellular uptake rate of prodrugs and on the conversion rate to MKH in cells. Intracellular delivery of MKH after MK-4 administration was dependent on the reductive activation of MK-4 to MKH. The ratio of AUC_MKO_/AUC_MK-4_ shown in Table 3 suggests the difference of delivery processes between MKH prodrug and MK-4. These ratios after MKH prodrugs administration were clearly higher than those obtained after MK-4 administration, indicating that these esters are hydrolyzed to MKH and effectively converted to MKO in HaCaT cells.

**Figure 8:**
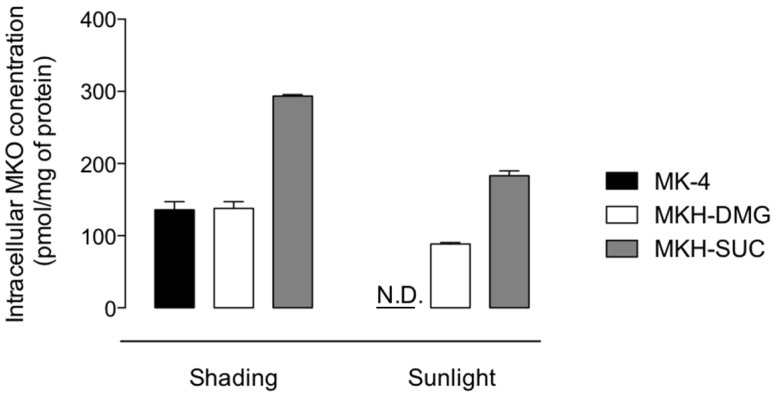
MKO levels in HaCaT cells after 48 h of treatment with previously sunlight-irradiated MK-4 or MKH derivatives. Shading sample cells were treated with each compound with no sunlight exposure. Sunlight sample cells were treated with each compound irradiated with sunlight (12,000 lx) for 3 h. Data represent mean ± SD (*n* = 3).

To confirm whether MKH prodrugs could successfully deliver MKH to dermal cells under sunlight, the intracellular level of MKO in HaCaT cells was determined following administration of MK-4 and MKH derivatives in the presence or absence of artificial sunlight irradiation. The intracellular MKO level after administration of MK-4 and irradiation with artificial sunlight was seriously reduced compared to that following MK-4 administration in shade (Figure 8). MKO levels were increased after administration of MKH-DMG and MKH-SUC in sunlight, but these were approximately 60% that of the cells treated with their respective compounds in shading conditions. These results clearly indicate that the MKH prodrugs (MKH-DMG and MKH-SUC) could deliver MKH into keratinocytes even under sunlight irradiation, but MK-4 could not. It is also possible that MK-4 chromenol, the photodegradation product of MK-4, could not reconvert to MK-4 or MKH in keratinocytes.

## 3. Discussion

The relative irradiance of artificial sunlight used in this study at wavelengths of 300 to 800 nm is approximately equal to that of natural sunlight. Thus, the high photostability of MKH derivatives compared with the quinone form of MK-4 suggests that MKH derivatives may be useful for skin application without photodegradation. A study of the vitamin K_1_ photoreaction by Hangarter et al. showed that charge transfer from the β,γ-double bond of the isoprenyl side chain to the quinone moiety initiates intramolecular proton transfer from the side chain, yielding the 1,3-quinone methide diradical in polar solvents and subsequently forms 1,2-quinone methide, which forms vitamin K_1_ chromenol [12]. The formation of MK-4 chromenol in ethanol following irradiation of MK-4 suggests that it is formed by the same mechanism. The bis-ester of MKH was hydrolyzed to MKH monoester by irradiation, which may also be explained by the formation of 1,3-quinone methide in a polar solvent that could then intramolecularly transfer from the side chain to the quinone moiety. MKH-DMG and MKH-SUC hydrolyses were accelerated only at 279 nm, confirming the hydrolyses of MKH-bis-ester to monoester in sunlight.

MK-4 degraded following a reaction rate-light wavelength relationship, in which exposure to shorter wavelengths resulted in faster degradation rates. However, Teraoka et al. showed that the curve of photodegradation rate of MK-4 against wavelength followed a bell bottom shape and that the fastest rate occurred at 430 nm [10]. In their study, MK-4 was solubilized in rapeseed oil, which shows large absorptivity at <300 nm. Thus, it is likely that irradiation energy at <300 nm was absorbed by rapeseed oil, resulting in reduced exposure of MK-4 to irradiation energy and producing the bell bottom profile.

Our results indicate that UVA irradiation of MK-4 accelerates singlet oxygen generation in aqueous solution, and increases intracellular ROS generation and cell toxicity in the HaCaT keratinocyte cell line. These results may be related to 1,3-quinone methide diradical formation by UVA irradiation of MK-4. Contrastingly, MKH derivatives avoided ROS formation and phototoxicity under UVA irradiation. Therefore, MKH derivatives would be candidates for effective, non-phototoxic use in skin application.

In order to act as an MKH prodrug, MKH derivatives must be converted to the parent drug in dermal cells after skin application. Our results clearly show that MKH derivatives and MK-4 increase intracellular MKO levels in the keratinocyte cell line we used, indicating that they could be used to deliver MKH into keratinocytes, where it then acts as a cofactor for GGCX. We previously showed that MKH-DMG and MKH-SUC reconvert to MKH in a reaction catalyzed by carboxyl esterase [17], which is present in dermal cells [19,20,21], so accelerated intracellular reconversion to a parent drug is possible. Although UVA-irradiated MK-4 did not increase intracellular MKO, UVA-irradiated MKH derivatives were able to.

MKH delivery with MKH-SUC into HaCaT cells is faster than that with MKH-DMG. A similar result was obtained while using hepatocellular carcinoma (HCC) cells in a previous study—the uptake and reconversion rates of MKH-SUC in HCC cells were faster than those of MKH-DMG [17]. Therefore, it can be presumed that the rapid and increased MKH delivery with MKH-SUC in HaCaT cells is due to the rapid uptake and fast reconversion rates.

In conclusion, MKH-DMG and MKH-SUC could act as MKH prodrugs for skin application without photoinstability and phototoxicity. This ideal strategy is a safer and more efficient way to deliver MKH than using the quinone form MK-4. Therefore, MKH prodrugs have the potential to be used for skin diseases which require the activity of vitamin K dependent proteins for treatment, even under direct sunlight irradiation. Further studies that can reflect the actual skin environment using human origin skin models such as 3-dimensional skin models or flesh skin tissue samples are needed.

## 4. Materials and Methods 

### 4.1. Chemicals

MK-4 was purchased from Seebio Biotech, Inc. (Shanghai, China). Menaquinone-4 epoxide (MKO) was kindly provided by Eisai Co., Ltd. (Tokyo, Japan). Menahydroquinone-4 1,4-bis-*N*,*N*-dimethylglycinate hydrochloride (MKH-DMG) and menahydroquinone-4 1,4-bis-hemisuccinate (MKH-SUC) were synthesized in our laboratory using previously reported methods [17]. Other chemicals were purchased from FUJIFILM Wako Pure Chemical Corporation (Osaka, Japan).

### 4.2. Cell Culture

Human epidermal keratinocyte cell line HaCaT was obtained from CLS Cell Lines Service GmbH (Eppelheim, Germany). Cells were maintained in DMEM (high glucose, CLS) with 10% FBS (Life Technologies, Carlsbad, CA, USA) and 1% penicillin/streptomycin (Life Technologies) at 37 °C under an atmosphere of 5% CO_2_.

### 4.3. Photostability

Solutions of MK-4 and MKH derivatives in ethanol (1 µM) were irradiated with artificial sunlight (SOLAX 100 W XC-100 B, Seric Ltd., Tokyo, Japan) at 12,000 lx in quartz cells. Illuminance was measured using a digital luminometer (LX-1108, Mother Tool). Irradiation samples (1 µM in ethanol) were irradiated with artificial sunlight at 12,000 lx, and with monochromatic light (279, 341, 373, 404, and 435 nm) using a multi-wavelength irradiation spectrometer (MM 3, Bunkoukeiki Co., Ltd., Tokyo, Japan). Irradiation intensity was measured using an irradiation energy measurement power meter (MM 3, Bunkoukeiki Co., Ltd.). Concentrations of MK-4, MK-4 chromenol, MKH-DMG, MKH-mono-NN-dimethyl glycinate (MKH-mono-DMG), MKH-SUC, and MKH-mono-succinate (MKH-mono-SUC) in each solution were analyzed by LC-MS/MS as described below (Section 4.9).

### 4.4. Singlet Oxygen Generation Assay

MK-4 and MKH derivatives were dissolved in a phosphate buffer (NaPB, pH 7.4) containing 0.2% polyoxyethylene hydrogenated castor oil 40 (HCO 40, obtained as a gift sample from Nikko Chemicals Co., Ltd., Osaka, Japan), 2% dimethyl sulfoxide, and 0.1% glycerol. The solutions were treated according to the singlet oxygen assay method (The Japanese Center for the Validation of Alternative Methods, JaCVAM) [22] and irradiated with UVA light from a CL-1000L UV Crosslinker (UVP, Upland, CA, USA). Absorbance was measured using an Infinite M200 PRO (Tecan Life Sciences, Zurich, Switzerland).

### 4.5. Intracellular ROS Generation Assay

Intracellular ROS generation was measured using 2′,7′-dichlorofluorescein diacetate (DCFH-DA; Invitrogen, Carlsbad, CA, USA) as a fluorescent probe. HaCaT cells were seeded at 1.0 × 10^5^ cells/well in 96-well plates and cultured overnight. Thereafter, cells were washed with PBS and incubated with 10 µM DCFH-DA solution in PBS at 37 °C for 1 h. Following incubation, test compound solutions in PBS described above were added to each well and re-incubated for 1 h. After incubation, cells were irradiated with UVA at an irradiation intensity of 15 J/cm^2^ and the fluorescence intensity (Ex: 485 nm, Em: 530 nm) was measured using an Infinite M200 PRO (Tecan Life Sciences).

### 4.6. Cell Viability Assay

HaCaT cells were seeded at 1.0 × 10^5^ cells/well in 96-well plates and cultured overnight. Thereafter, cells were washed with PBS, and test compound solutions in PBS described above were added to each well and re-incubated for 1 h. After incubation, cells were irradiated with UVA at an irradiation intensity of 15 J/cm^2^. After irradiation, cells were washed twice with PBS and replaced in culture medium. After 24 h incubation, cell viability was measured using a CellTiter-Glo^®^ Luminescent Cell Viability Assay (Promega, Madison, WI, USA).

### 4.7. Determination of Intracellular MKO Levels after Treatment With MK-4 and MKH Derivatives

HaCaT cells were seeded at 5.0 × 10^4^ cells/well in 24-well plates and allowed to attach for 48 h. Cells were cultured in medium containing 5 µM MK-4, MKH-DMG, or MKH-SUC, then medium was removed and cells were washed twice with PBS. Cells were collected in 500 µL of PBS and sonicated. Cell homogenates were combined with an equal volume of methanol and three times volume of *n*-hexane, vortexed for 2 min, and centrifuged at 1750× *g* for 10 min. The organic layer was evaporated under N_2_ gas. The residue was reconstituted with 20 μL of methanol, and subjected to LC-MS/MS, as described below. The protein concentration of the cell homogenate was determined using a BCA protein assay kit (Thermo Fisher Scientific, Waltham, MA, USA).

### 4.8. Determination of Intracellular MKO Levels After Treatment With Sunlight-Irradiated MK-4 and MKH Derivatives

Solutions of 200 µM MK-4, MKH-DMG, or MKH-SUC in ethanol were irradiated with artificial sunlight for 3 h at 12,000 lx in quartz cells. After irradiation, ethanol was evaporated under N_2_ gas. Residues were re-dissolved in a volume of medium equal to the evaporated ethanol, diluted 40-fold, and added to the cells for up to 48 h. Measurement of intracellular MKO was carried out using the same method as described above (Section 4.7).

### 4.9. LC-MS/MS

LC-MS/MS was performed using an LCMS-8050 Liquid Chromatograph Mass Spectrometer (Shimadzu, Kyoto, Japan) and Shimadzu HPLC System (system controller (CBM-20A), pump (LD-20AD), degasser (DGU-20As), UV detector (SPD-20A), and auto injector (SIL-20AC HT)). Separations were performed on a CAPCELL PAK C18 UG120 (3 μm, 2.0 mm × 100 mm, Shiseido Co., Ltd., Tokyo, Japan) using a mobile phase of 10 mmol/L ammonium acetate and 0.1% acetic acid in methanol and water (97:3) at a flow rate of 0.4 mL/min. Column temperature was maintained at 40 °C. The mass spectrometer was equipped with an electrospray ionization and was run in positive ion mode. Identification and quantitation were based on MS/MS-multiple reaction monitoring mode using transition ions as follows: *m*/*z* 445 → 187 for the [M+H]^+^ MK-4 adduct, *m*/*z* 445 → 187 for the [M + H]^+^ chromenol adduct, 619 → 58 for the [M + H]^+^ MKH-DMG adduct, *m*/*z* 532 → 58 for the [M + H]^+^ MKH-mono-DMG adduct, *m*/*z* 664 → 187 for the [M + H]^+^ MKH-SUC adduct, *m*/*z* 564 → 187 for the [M + H]^+^ MKH-mono-SUC adduct, and *m*/*z* 461 → 81 for the [M + H]^+^ MKO adduct. Retention times were: MK-4, 3.3 min; MK-4 chromenol, 2.0 min; MKH-DMG, 1.7 min; MKH-mono-DMG, 1.6 min; MKH-SUC, 1.1 min; MKH-mono-SUC, 1.2 min; and MKO, 2.5 min.

### 4.10. Statistical Analysis

Statistical significance was determined using Dunnett’s test. *p* < 0.05 was considered statistically significant. Data were analyzed using GraphPad Prism 6 (GraphPad Software).

## Figures and Tables

**Table 1:** The apparent first order rate constants (*k*) and half-lives (*t*_1/2_) of degradation of MK-4 and MKH derivatives in ethanol under irradiation with artificial sunlight (12,000 lx) at 25 °C.

Compound ^a^	Irradiation Conditions	*k* (h^−1^)	*t*_1/2_ (h)
MK-4	Sunlight	8.239	0.084
MKH-DMG	Sunlight	0.167	4.150
MKH-SUC	Sunlight	2.796	0.248
	Shading ^b^	0.883	0.785

^a^ Initial concentrations were 1 µM in ethanol. ^b^ During irradiation, compound was covered with aluminum foil for shading.

**Table 2:** The rate constants (*k*) and half-lives (*E*_1/2_) of degradation of MK-4 and MKH derivatives in ethanol under different irradiation intensities of monochromatic light at 25 °C.

Compound ^a^	Wavelength (nm)	*k* (J^−1^·cm^2^)	*E*_1/2_ (J^−1^·cm^2^)
MK-4	279	0.533	1.301
	341	0.422	1.643
	373	0.151	4.583
	404	0.049	15.800
	435	0.035	19.738
MKH-DMG	279	0.146	4.750
	341	- ^b^	- ^b^
	373	- ^b^	- ^b^
	404	- ^b^	- ^b^
	435	- ^b^	- ^b^
MKH-SUC	279	0.110	6.323
	341	0.069	10.036
	373	0.059	11.792
	404	0.061	11.296
	435	0.068	10.253

^a^ Initial concentrations were 1 µM in ethanol. ^b^ No decomposition.

**Table 3:** The area under curve over 72 h (AUC_MKO (0–72 h)_) of MKO and (AUC_MK-4 (0–72 h)_) of MK-4 concentrations and the AUC ratio of MKO to MK-4 (AUC_MKO_/AUC_MK-4_) over time in HaCaT cells treated with MK-4 or MKH derivatives.

Compound ^a^	AUC_MKO (0–72 h)_ (nmol·h/mg of Protein)	AUC_MK-4 (0–72 h)_ (nmol·h/mg of Protein)	AUC_MKO_/AUC_MK-4_
MK-4	10.543 ± 0.795	5.628 ± 0.698	1.873 ± 0.272
MKH-DMG	10.786 ± 1.696	1.878 ± 0.088	5.743 ± 0.942
MKH-SUC	17.304 ± 1.068	2.316 ± 0.095	7.471 ± 0.554

^a^ Cells were treated with medium containing 5 µM MK-4, MKH-DMG, and MKH-SUC. Data represent mean ± SD (*n* = 3).

## Supplementary Materials

Supplementary materials can be found at https://www.mdpi.com/1422-0067/20/10/2548/s1.

## Abbreviations

MKH	menahydroquinone-4
MK-4	menaquinone-4
MKC	menaquinone-4 chromenol
MKH-DMG	menahydroquinone-4 1,4-bis-*N*,*N*-dimethylglycinate hydrochloride
MKH-mono-DMG	menahydroquinone-4 1-mono *N*,*N*-dimethylglycinate hydrochloride menahydroquinone-4 4-mono *N*,*N*-dimethylglycinate hydrochloride
MKH-SUC	menahydroquinone-4 1,4-bis-hemisuccinate
MKH-mono-SUC	menahydroquinone-4 1-mono hemisuccinate menahydroquinone-4 4-mono hemisuccinate
MKO	menaquinone-4 epoxide
GGCX	γ-glutamyl carboxylase
VKORC1	vitamin K epoxide reductase complex subunit 1
VKORC1L1	vitamin K epoxide reductase complex subunit 1 like 1
ROS	reactive oxygen species
DCFH-DA	2′,7′-dichlorofluorescein diacetate
AUC	area under the concentration versus time curve
MRM	MS/MS-multiple reaction monitoring mode
