# Supplementary material for: Prodrugs for Skin Delivery of Menahydroquinone-4, an Active Form of Vitamin K2(20), Could Overcome the Photoinstability and Phototoxicity of Vitamin K2(20)
Source: Int J Mol Sci. 2019 May 24;20(10):2548. doi: 10.3390/ijms20102548 (PMC6566782; doi:10.3390/ijms20102548)
Supplement: Supplementary file 1 [file ijms-20-02548-s001.pdf]

Supplemental Figure

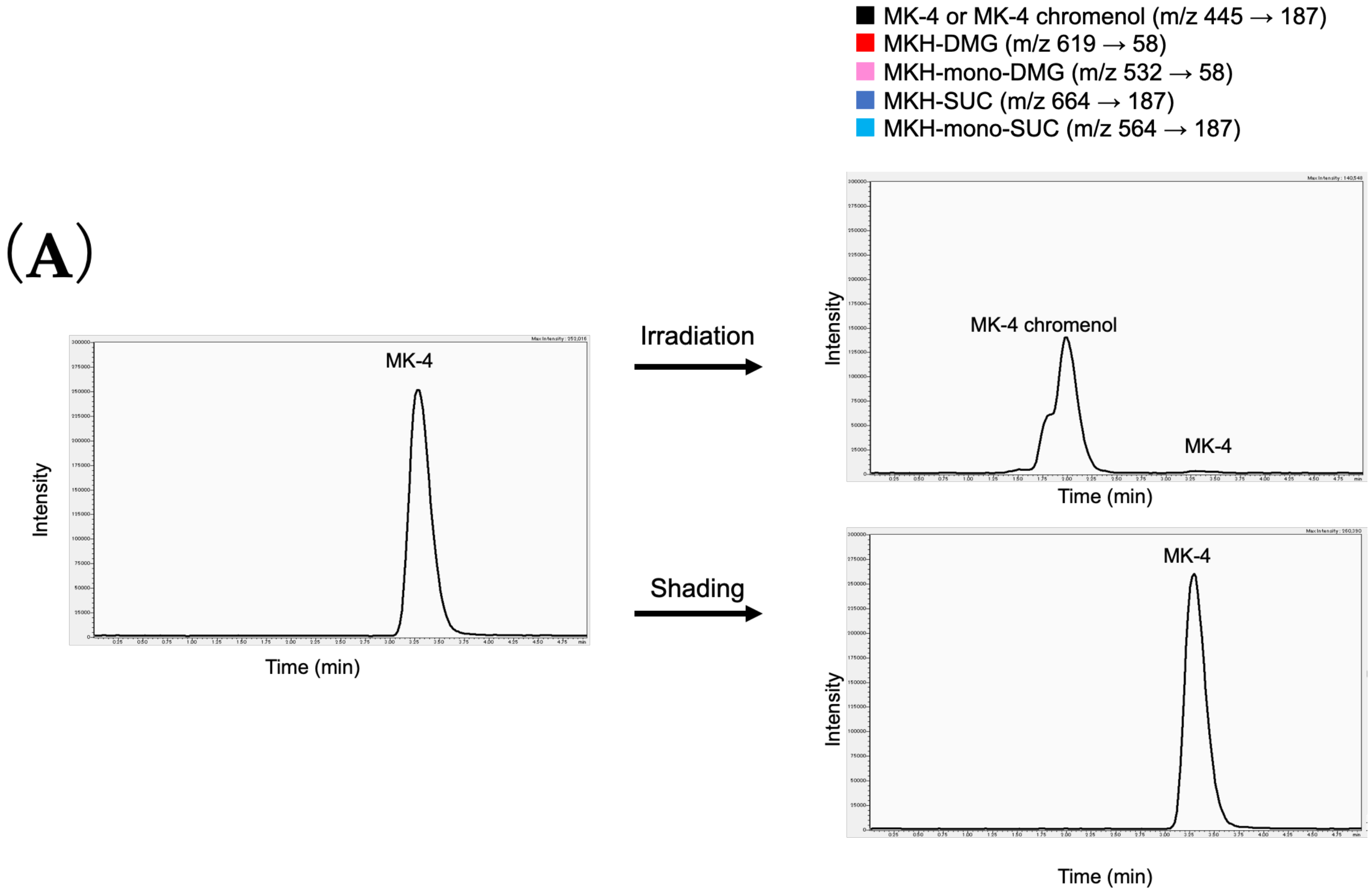

(B)

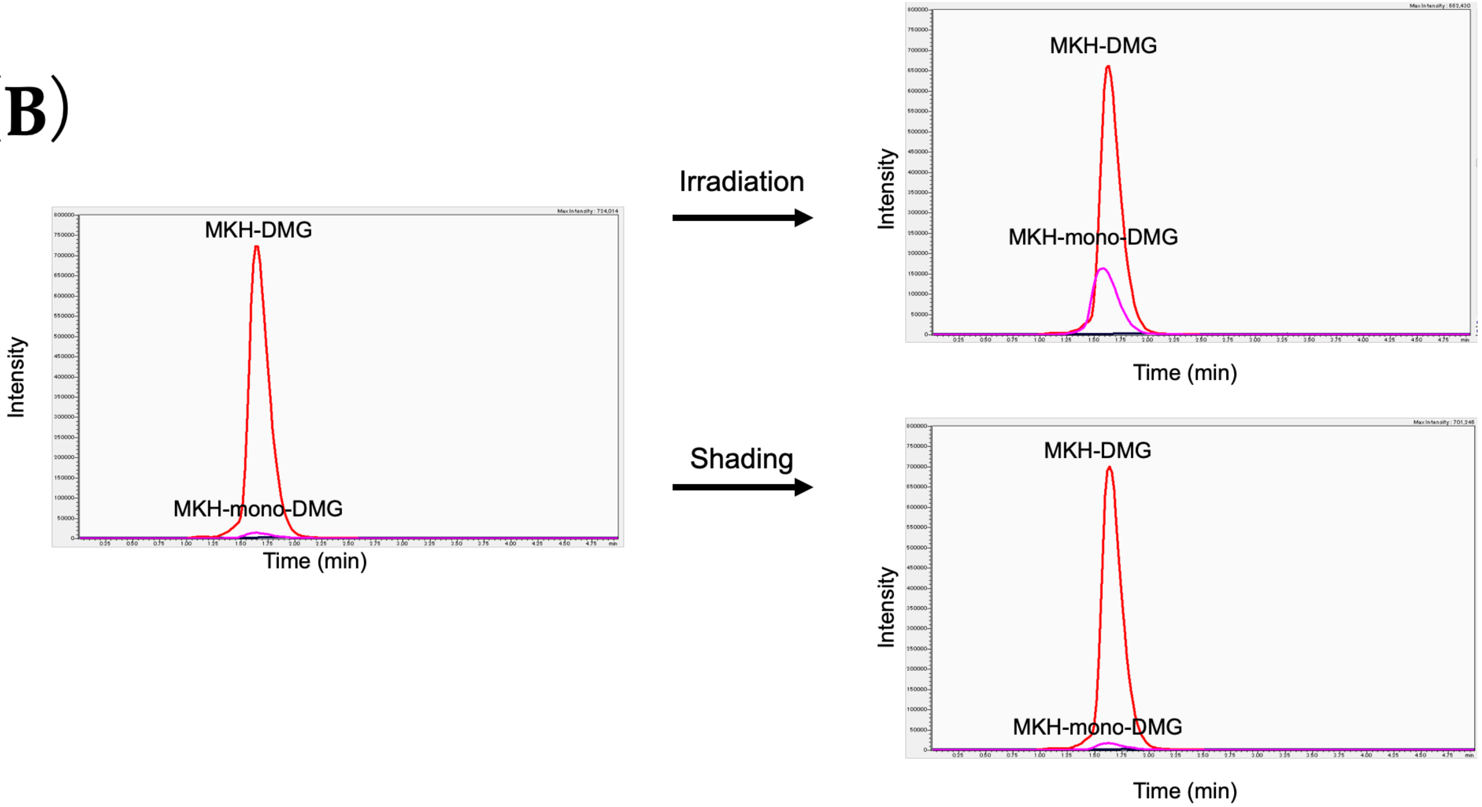
MKH-DMG
MKH-mono-DMG
Intensity
Time (min)
Irradiation
MKH-DMG
MKH-mono-DMG
Intensity
Time (min)
Shading
MKH-DMG
MKH-mono-DMG
Intensity
Time (min)

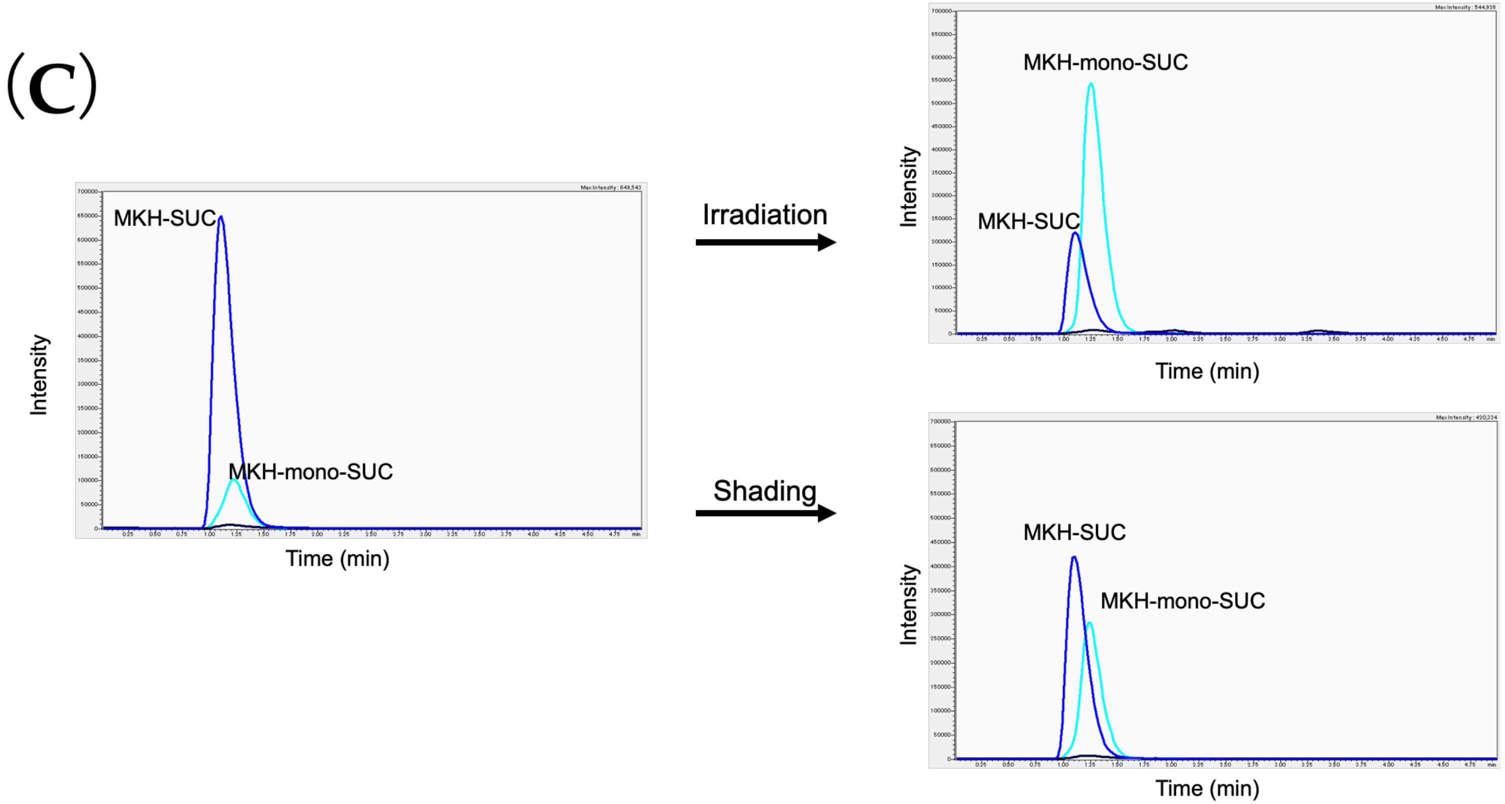


**Figure S1. Typical LC-MS/MS MRM chromatograms of MK-4 and MKH derivatives irradiated with artificial sunlight.**

(**A**) MK-4, (**B**) MKH-DMG and (**C**) MKH-SUC with and without shading in ethanol (1 μM). Irradiated with artificial sunlight (12000 lx) for 30 min at 25 °C.
